# Cardiotoxicity from neoadjuvant targeted treatment for breast cancer prior to surgery

**DOI:** 10.3389/fcvm.2023.1078135

**Published:** 2023-02-22

**Authors:** Yihua Liu, Li Zheng, Xingjuan Cai, Xiaojun Zhang, Yang Ye

**Affiliations:** ^1^Department of Breast Surgery, Xiyuan Hospital, China Academy of Chinese Medical Sciences, Beijing, China; ^2^Department of Traditional Chinese Medicine, Peking University Third Hospital, Beijing, China

**Keywords:** breast cancer, targeted therapy, review, mechanism, cardiotoxicity

## Abstract

Cancer treatment has been gradually shifting from non-specific cytotoxic agents to molecularly targeted drugs. Breast cancer (BC), a malignant tumor with one of the highest incidence worldwide, has seen a rapid development in terms of targeted therapies, leading to a radical change in the treatment paradigm. However, the use of targeted drugs is accompanied by an increasing rate of deaths due to non-tumor-related causes in BC patients, with cardiovascular complications as the most common cause. Cardiovascular toxicity during antitumor therapy has become a high-risk factor for survival in BC patients. Targeted drug-induced cardiotoxicity exerts a wide range of effects on cardiac structure and function, including conduction disturbances, QT interval prolongation, impaired myocardial contractility, myocardial fibrosis, and hypertrophy, resulting in various clinical manifestations, e.g., arrhythmias, cardiomyopathy, heart failure, and even sudden death. In adult patients, the incidence of antitumor targeted drug-induced cardiotoxicity can reach 50%, and current preclinical evaluation tools are often insufficiently effective in predicting clinical cardiotoxicity. Herein, we reviewed the current status of the occurrence, causative mechanisms, monitoring methods, and progress in the prevention and treatment of cardiotoxicity associated with preoperative neoadjuvant targeted therapy for BC. It supplements the absence of relevant review on the latest research progress of preoperative neoadjuvant targeted therapy for cardiotoxicity, with a view to providing more reference for clinical treatment of BC patients.

## Introduction

According to the Global Cancer Registry 2020, breast cancer (BC) is one of the most prevalent malignancies worldwide, accounting for 11.7% of all cancer cases. Approximately 2.26 million new BC cases and 685,000 deaths were reported in 2020. In China, 420,000 new BC cases were reported in 2020, which is the highest number of new malignant tumor cases in Chinese women, accounting for 19.9% of all malignant tumors in women (1). The development of targeted drugs has led to a dramatic change in the treatment paradigm for BC, which stems from the advances in understanding of the biological mechanisms of tumorigenesis and cancer progression (2). Both domestic and international guidelines recommend neoadjuvant therapy combined with localized surgical excision as the first-line targeted therapy in patients with human epidermal growth factor receptor 2 (HER2)-positive BC patients (1, 3). As a result of multiple clinical trials and novel treatment concepts in BC, the therapy paradigm has shifted from chemotherapy alone to neoadjuvant therapies, such as anti-HER2-targeted therapy combined with chemotherapy and neoadjuvant endocrine therapy (4, 5). In particular, neoadjuvant targeted therapy is increasingly used in clinical practice due to its high efficacy in reducing the stage of primary breast tumors and involved axillary lymph nodes. This type of therapy is also associated with high drug sensitivity and improved breast conservation rates (6–8).

As an important treatment option for BC, neoadjuvant therapy is no longer indicated only for locally advanced BC. Pathological complete response (pCR) is significantly associated with improved overall survival and disease-free survival after neoadjuvant therapy, and is the most objective and efficient indicator of the efficacy of neoadjuvant therapy (9). HER2-positive BCs account for 20–30% of all BC types (10, 11), and the amplification and overexpression of the HER2 gene is strongly associated with high degree of malignancy, insensitivity to conventional chemotherapy, and poor clinical prognosis in BC (12, 13). The most commonly used preoperative neoadjuvant targeted therapies for BC are anti-HER2 monoclonal antibodies, mainly trastuzumab and pertuzumab (6, 14, 15). In recent years, new targeted drugs have emerged, such as (1) multi-targeted small-molecule tyrosine kinase inhibitors (TKI), e.g., lapatinib (16); (2) inhibitors of cyclin-dependent kinases 4/6 (CDK4/6), e.g., reboxetine (17); and (3) anti-vascular endothelial growth factor (VEGF) monoclonal antibodies, e.g., bevacizumab (18, 19), offering new options for the treatment of HER2-positive BC. The HER2 gene is also present in human ventricular myocytes, and its sustained expression is important for several biological processes, such as myocardial fiber trabeculae formation, cardiac morphogenesis, cardiomyocyte survival under injury/stress conditions, and cardiomyocyte differentiation and maturation (20–22). Anti-HER2 targeted therapy blocks neuregulin, an important regulator of cardiovascular homeostasis, involved in cardiomyocyte differentiation *via* HER2, resulting in the inactivation of various intracellular pathways that promote cell proliferation, resistance to apoptosis, and regulate mechanical properties of cells. These pathways involve mitogen-activated protein kinase, phosphoinositide 3-kinase (PI3K)/protein kinase B (AKT)/mammalian target of rapamycin (mTOR), signal transducer and activator of transcription, and protein kinase C (PKC) signaling, among others (23–25). The dysfunction of these pathways, in turn, leads to an increased sensitivity of ventricular myocytes to injury and stress (e.g., increased intracellular oxidative stress and volume loading caused by anthracycline chemotherapeutics) and overexpression of apoptotic proteins (26). Ultimately, this can lead to the development of cancer therapy-related cardiac dysfunction (CTRCD). The 2022 ESC Oncology Cardiology Guidelines precisely define cardiovascular toxicity associated with cancer therapy. In addition to the associated clinical symptoms, asymptomatic CTRCD is defined as left ventricular ejection fraction (LVEF) ≥50% and a new relative decline in global longitudinal strain (GLS) of >15% from baseline. Immune checkpoint inhibitor (ICI)-associated myocarditis is primarily diagnosed on the basis of endomyocardial heart machine biopsies with multifocal inflammatory cell infiltration and significant myocardial cell necrosis on light microscopy and cardiovascular magnetic resonance (CMR) imaging. A prolonged QT interval is defined on electrocardiogram (ECG) as a corrected QT interval using Fridericia correction of >500 ms (27). This cardiotoxicity of anti-HER2 targeted therapies has become a major obstacle to their clinical use. This paper describes the causative mechanisms, risk factors, monitoring tools, and interventions for cardiovascular toxicity associated with preoperative neoadjuvant targeted therapy in BC patients based on recent medical evidence.

## Neoadjuvant targeted therapy strategy for BC

Neoadjuvant targeted therapy has become the most effective treatment modality for locally advanced BC and inflammatory BC, as well as the preferred treatment modality for the majority of stage II and III triple-negative BC (TNBC) and HER2-positive BC cases (28). In HER2-positive patients, the 2021 St. Gallen Expert Consensus recommends a dual-targeted regimen of TCb (paclitaxel in combination with platinum) and HP (trastuzumab monoclonal antibody in combination with pertuzumab) for the neoadjuvant targeted therapy phase; however, HER2-positive patients with cT1cN0 and some with cT2N0 disease can switch to monotherapy after achieving pCR with dual-targeted therapy (29).

Long-term follow-up studies indicate that pCR in TNBC and HER2-positive BC is significantly associated with improved patient survival (30). Therefore, patients who do not achieve pCR after neoadjuvant targeted therapy should be administered intensive therapy promptly to better their prognosis. The results of the KATHERINE clinical trial suggest that in HER-2-positive patients who did not achieve pCR after standard neoadjuvant targeted therapy regimens, the use of T-DM1 in the adjuvant phase increased the 3-year infiltrative disease-free survival (iDFS) and distant recurrence-free survival by 11.3 and 6.7%, respectively (31). For HER2-positive patients who have not achieved pCR, intensive T-DM1 therapy is recommended in the postoperative adjuvant phase.

The results of the EXTENET study showed that for hormone receptor (HR)-positive or HER2-positive patients who did not achieve pCR after neoadjuvant targeted therapy, one year of lenvatinib within 2 years after trastuzumab monoclonal antibody treatment improved the 5-year iDFS by 7.4%, suggesting the potential benefit of lenvatinib for adjuvant intensive treatment strategies after neoadjuvant targeted therapy for HR-positive or HER2-positive patients (32). The results of the CREATE-X study suggested that in HER2-negative patients who did not achieve pCR after neoadjuvant targeted therapy, the addition of capecitabine improved the prognosis, especially in TNBC patients, and increased the 5-year disease-free survival by 13.7%.

## Mechanisms of cardiovascular toxicity associated with targeted therapeutic agents for BC

### Anti-HER2 monoclonal antibodies

Trastuzumab is a humanized monoclonal antibody targeting HER2 (33). By binding to HER2 on the surface of tumor cells, trastuzumab blocks the binding of human epidermal growth factor to HER2, thereby affecting the transmission of tumor cell growth signals for controlled tumor proliferation and delayed recurrence and metastasis (34, 35). Trastuzumab is primarily used in HER2-positive BC patients, usually in combination with docetaxel or paclitaxel (36). Pertuzumab is another monoclonal antibody targeting HER2, acting on different extracellular structural domains of HER2 than trastuzumab, inhibiting HER2 heterodimerization and, consequently, receptor signaling (37). In clinical practice, pertuzumab is often used in combination with trastuzumab to treat HER2-positive BC patients (38). This combination enhances the blocking effect of the downstream signaling; at the same time, they can jointly exert antibody-dependent cell-mediated cytotoxic effects, thus enhancing the immune-synergistic outcome (39, 40).

**Figure 1:**
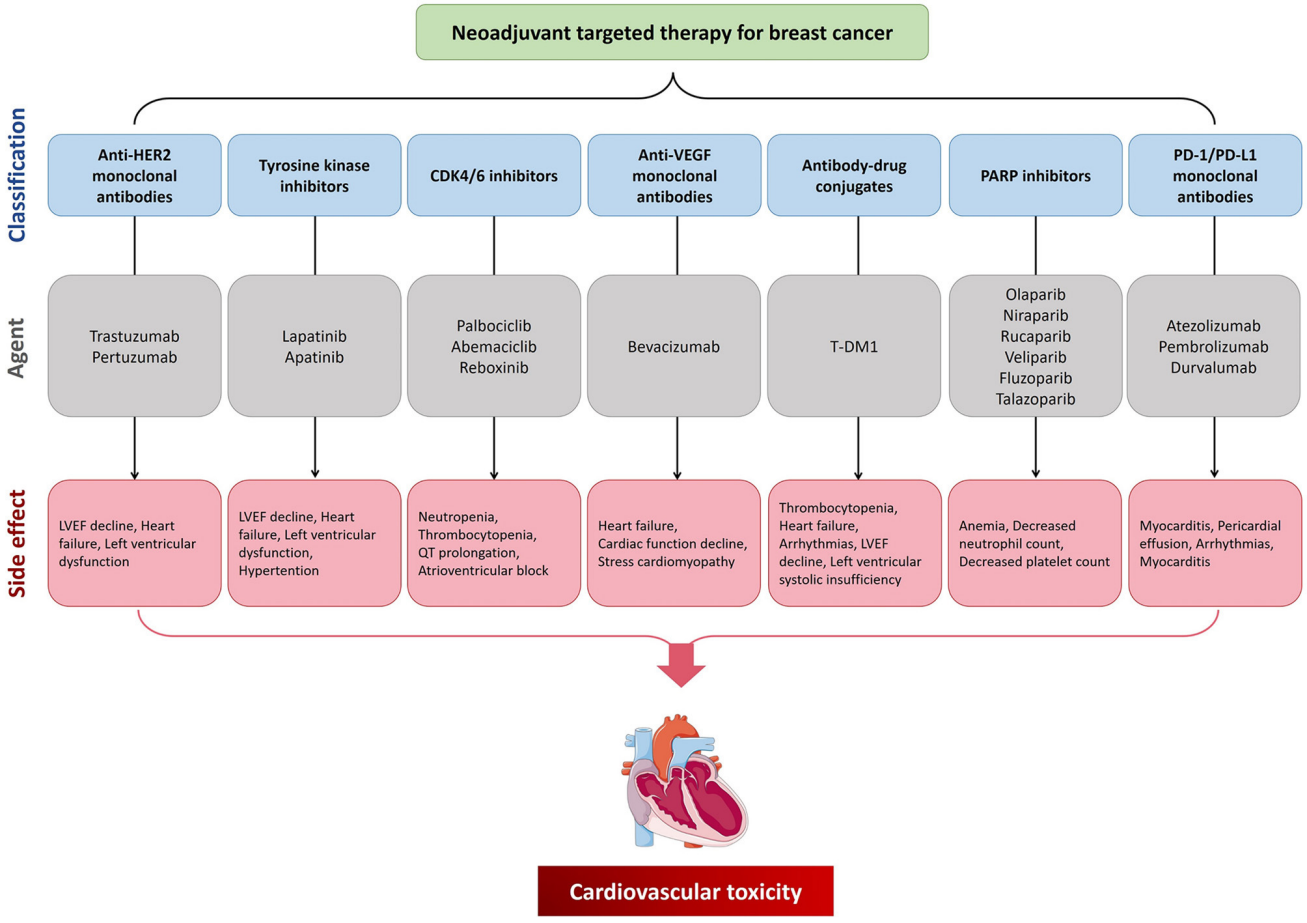
The cardiovascular toxicity of neoadjuvant targeting treatment for breast cancer. CDK4/6, cyclin-dependent kinases 4/6; HER2, human epidermal growth factor receptor 2; LVEF, left ventricular ejection fraction; PARP, poly adenosine diphosphate ribose polymerase; PD-1, programmed cell death protein 1; PD-L1, programmed cell death 1 ligand 1; VEGF, vascular endothelial growth factor.

Numerous studies have shown that trastuzumab increases the risk of cardiovascular toxicity (Table 1; Figure 1). In five phase III clinical trials of primary trastuzumab adjuvant therapy, a decrease in LVEF of ≥10% or 15% from baseline values was observed in 3% to 34% of patients, while the incidence of grade III–IV congestive heart failure by NYHA cardiac functional grading was 0–3.9% in the targeted therapy group and 0–1.3% in the untreated group (41, 42). In addition, the incidence of heart failure in HER2-positive metastatic BC patients treated with trastuzumab in combination with anthracyclines was 27%, whereas that of serious cardiovascular adverse events in the anthracycline only group was just 8%. This suggests that cardiovascular toxicity is a significant adverse effect of anthracyclines combined with trastuzumab (40, 43, 44). The NeoSphere trial demonstrated that patients receiving dual-target (trastuzumab combined with pertuzumab) neoadjuvant therapy had no significant increase in the incidence of left ventricular dysfunction compared with patients receiving single neoadjuvant targeted therapy, and the difference in the incidence of LVEF decreased by more than 10–15% from baseline or post-treatment LVEF <50% was not statistically significant (39, 45). In the CLEOPATRA trial, the incidence of cardiovascular adverse events was 14.5 and 16.4% in HER2-postive metastatic BC patients receiving first-line dual-targeted therapy or trastuzumab only, respectively. The incidence rates of grade III or higher cardiovascular adverse events (left ventricular systolic dysfunction was the most common) were 1.5 and 3.8%, respectively (46, 47).

**Table 1:** Mechanisms of cardiovascular toxicity associated with neoadjuvant targeting agents for breast cancer.

**Agent**	**Indication**	**Mechanism of cardiovascular toxicity**
***Anti-HER2 monoclonal antibody***		
Trastuzumab	HER2-positive breast cancer	Induce ROS overproduction and apoptosis of cardiomyocytes
Pertuzumab		
**Tyrosine kinase inhibitor**		
Lapatinib	HER2-postive progressive or metastatic breast cancer	Inhibit NO synthase and induce ROS overproduction
Apatinib		
**CDK4/6 inhibitor**		
Palbociclib	ER-positive/HER2-negative breast cancer	Affect QT interval-related genes and potassium and sodium channels
Abemaciclib		
Reboxinib		
**Anti-VEGF monoclonal antibody**		
Bevacizumab	Metastatic breast cancer; Triple negative breast cancer	Reduce capillary density and induce endothelial dysfunction
**Antibody-drug conjugate**		
T-DM1	HER2-positive breast cancer	Induce ROS overproduction and apoptosis of cardiomyocytes
**PARP inhibitor**		
Olapari	HER2-negative breast cancer with BRCA 1/2 mutation	Target and off-target effects
Niraparib		
Rucaparib		
Veliparib		
Fluzoparib		
Talazoparib		
**PD-1/PD-L1 monoclonal antibody**		
Atezolizumab	Triple negative breast cancer	Attack antigens of cardiomyocytes
Pembrolizumab		
Durvalumab		

DM1, mertansine; ER, estrogen receptor; HER2, human epidermal growth factor receptor 2; NO, nitric oxide; PARP, poly adenosine diphosphate ribose polymerase; PD-1, programmed cell death protein 1; PD-L1, programmed cell death 1 ligand 1; ROS, reactive oxygen species; VEGF, vascular endothelial growth factor.

Although the mechanism behind these cardiac effects is unclear, studies have suggested that it may be associated with the following processes. (1) Abnormalities in the HER2 signaling pathway. HER2 is expressed on cardiac myocytes, and the HER2 pathway stabilizes the fibrous structure of tissues through a series of signal transduction pathways, thereby inhibiting apoptosis, which can maintain cell survival by reducing the level of organic oxygen. However, trastuzumab inhibits HER2 signaling by binding to HER2, leading to excessive accumulation of reactive oxygen species (ROS) and destruction of cardiomyocytes in the presence of excess organic oxygen species, leading even to cardiac insufficiency. Thus, trastuzumab-related cardiovascular toxicity is an important clinical challenge (33, 48–50). (2) Abnormalities in neuromodulatory protein pathways. Under physiological conditions, the coronary microvascular endothelial cells and endocardium release neuromodulatory proteins (HER4), which boost proliferation pathways after dimerization with HER2. These pathways increase ROS synthesis inhibition pathways and maintain cellular integrity by reducing apoptosis, whereas anti-HER2 drugs disrupt the associated signaling, resulting in excessive production of ROS, which in turn impairs the integrity of cardiac vascular endothelial cells, affects myocardial blood supply, and weakens cardiomyocyte protection (51, 52). (3) Mitochondrial dysfunction. Mitochondria are physiologically important for maintaining the metabolic balance of cardiomyocytes and the homeostasis of the intracellular environment, as well as for regulating the cell growth cycle. Trastuzumab can impair the normal oxidative defense function of mitochondria in cardiomyocytes and induce massive accumulation of mitochondrial ROS, leading to the overexpression of the apoptotic protein Bcl-2 associated X protein (BAX), which in turn activates the mitochondrial apoptotic pathway, resulting in the loss of mitochondrial physiological function and opening of the mitochondrial permeability transition pores. This leads to apoptosis or necrosis of cardiomyocytes, ultimately resulting in cardiac tissue dysfunctions (53–55).

Multiple clinical studies have shown that trastuzumab triggers cardiovascular toxicity, with some degree of reversible damage (type II cardiovascular toxicity). Eight-year results from HERA trial suggested that the incidence of trastuzumab-induced cardiovascular events remained consistently low. Following treatment termination in patients with cardiovascular events in the 1-year treatment group, 79.5% of patients met the criteria for early recovery of cardiac function (56). The mechanism of reversibility is most likely related to the restoration of the HER2 pathway after trastuzumab discontinuation (57).

### Novel TKIs

Lapatinib is the first TKI approved for the treatment of HER2-postive advanced BC (58), which effectively inhibits phosphorylation of the epidermal growth factor receptor, blocks the downstream transmission of mitogenic signaling pathways, arrests tumor cell proliferation and differentiation, and promotes cell apoptosis (59). Lapatinib is currently approved for use in combination with capecitabine in advanced or metastatic BC patients who have failed to respond to anthracycline, paclitaxel, and trastuzumab treatments. A meta-analysis showed that most of the cardiovascular toxicity in clinical trial patients on lapatinib, regardless of whether anthracycline or trastuzumab has been used in the past, was only reflected by asymptomatic reduction of LVEF, which was largely reversible when the drug was discontinued (60). Numerous clinical phase I and II trials evaluating the safety of lapatinib in patients with progressive or metastatic HER2 BC have shown that the incidence of left ventricular dysfunction ranges from 0 to 26.6%, with a decrease in LVEF of ≥20% or a post-treatment assessment of LVEF <50%, ranging from 0 to 12.8% (61). However, it should be emphasized that most of these patients had received prior anthracycline-based chemotherapy or trastuzumab, and only a small percentage of patients received first-line lapatinib treatment. Another study analyzed the cardiovascular safety of continuing trastuzumab in combination with lapatinib in stage IV HER-2+ BC patients whose disease progressed during trastuzumab treatment and showed that the incidence of asymptomatic LVEF decline and congestive heart failure was lower in the combination group (2 and 3.4%, respectively) than in the lapatinib-only group (0.7 and 1.4%, respectively) (62). In a phase III clinical trial, locally progressive or metastatic HER2-postive BC patients who had disease progression after prior treatment with anthracyclines, paclitaxel, and trastuzumab and whose LVEF was within the normal range prior to enrollment received lapatinib + capecitabine and maintenance capecitabine alone, respectively; cardiovascular events (defined as asymptomatic heart failure or a ≥20% decrease in LVEF from baseline) remained low in both groups, i.e., at 2.45 and 0.62%, respectively (63).

Apatinib is a novel small-molecule vascular endothelial growth factor receptor (VEGFR) TKI, developed independently in China, that can control tumor growth and progression by inhibiting VEGFR and thus tumor angiogenesis (64, 65). Currently, it is widely used in clinical practice. Hypertension is one of the main adverse effects of this drug observed in clinical studies (only as isolated cases) (66–68). The mechanisms of hypertension caused by apatinib may include: (1) downregulation of NO synthase expression and decreased NO release, (2) sparse microvascular network, (3) increased secretion of vasoconstriction stimulants, (4) increased synthesis of ROS, and (5) renal insufficiency (69).

### CDK4/6 inhibitors

HR-positive BC is the most common type of BC worldwide. In recent years, CDK4/6 inhibitors have shown large benefits in HR-positive patients, and CDK4/6 inhibitor monotherapy combined with endocrine therapy has been used for the treatment of advanced BC. Since the U.S. Food and Drug Administration (FDA) approval of palbociclib in 2015, CDK4/6 inhibitors have been the first-line treatment option for patients with metastatic HR-positive/HER2-negative phenotypes (70). CDK4/6 inhibitors can block tumor cells in the first phase of the cell cycle (G1 phase) by binding to cell cycle proteins to promote the mid-phase transition, initiate DNA synthesis, and regulate cell transcription (71). Palbociclib, abemaciclib, and reboxinib are specific CDK4/6 inhibitors, and all three drugs are used in clinical studies as monotherapy or in combination with other drugs for the treatment of many types of tumors. Palbociclib is the first highly specific CDK4/6 inhibitor. A single-arm phase II study enrolling 37 patients with Rb-positive metastatic BC showed that of the 84% of ER-positive/HER2-negative BC patients, two had partial remission and five had stable disease for more than 6 months. In BC models, when palbociclib was combined with trastuzumab or tamoxifen, these drugs had a synergistic inhibitory effect on HER gene amplification and ER-positive cell proliferation; the same synergistic effect was observed when palbociclib was combined with endocrine therapy (72). Significant adverse events in early clinical trials often include neutropenia and thrombocytopenia owing to the dependence of bone marrow progenitor cells on CDK6 (73). Abemaciclib is the only CDK4/6 inhibitor approved for monotherapy (74) and has shown broader inhibition efficacy compared to other CDK4/6 inhibitors. Abemaciclib monotherapy was tested in the phase II single-arm MONARCH-1 study, which reported an objective remission rate of 19.7%, median progression-free survival (PFS) of 6 months, and median overall survival of 17.7 months (75). Additionally, clinical studies have shown that it can be used alone or in combination with gemcitabine (72). Owing to its broad inhibitory effects, the drug can induce a variety of side effects, with fatigue as a dose-limiting toxicity and diarrhea as a common adverse effect (74). Cardiovascular side effects are uncommon with CDK4/6 inhibitors and include only QT prolongation, which occurs at a low rate. Two cases of new onset 2nd degree type 2 atrioventricular block requiring permanent cardiac pacing in metastatic BC patients treated with reboxinib or abemaciclib have been recently reported (76). In 2017, the FDA approved reboxinib and aromatase inhibitors together as first-line endocrine therapy in postmenopausal women with advanced or metastatic BC that is HR-positive and HER2 negative (77). Reboxinib-induced cardiovascular toxicity mainly manifests as QT interval prolongation. In a study including 668 postmenopausal patients with HR+, HER2- recurrent, or metastatic BC who were evaluated for PFS with the combination of reboxinib and letrozole, 3.6% subjects showed prolonged QT intervals (78). The mechanism may involve: (1) abnormal expression of genes related to long QT syndrome (KCNH2, SCN5A, SNTA1, etc.). The reboxinib-induced long QT interval can be attributed to the regulation of the expression of one or several related genes. Microarray analysis of human leukemia cell lines revealed that three LQT-syndrome-related genes, KCNH2, SCN5A, and SNTA1, were differentially expressed in cells after treatment with reboxinib, showing a decrease in KCNH2 and an increase in SCN5A and SNTA1 (79). Another possible explanation could involve (2) alteration of potassium and sodium channels. It has been reported that drug-induced QT interval prolongation is caused by a blockade of potassium channels encoded by the human ether-go-go-related gene (hERG) (80). In a study comparing the safety of palbociclib and reboxinib, it was shown that reboxinib caused hERG inhibition (81).

### Anti-VEGF monoclonal antibodies

Bevacizumab is a recombinant humanized IGg1 monoclonal antibody that exerts antitumor effects by inhibiting the biological activity of VEGF and blocking tumor angiogenesis (82). The role of bevacizumab in BC has been controversial, and three major clinical studies, the E2100, AVADO, and RIBBON-1 studies, showed that bevacizumab prolonged PFS in patients, but there was no difference in overall survival (OS). The European Medicines Agency has approved bevacizumab in combination with paclitaxel as a first-line treatment for metastatic BC. A combination of these three clinical trials found that in triple-negative BC, the objective response rate (ORR) of chemotherapy in combination with bevacizumab increased by nearly 20%, and PFS was significantly prolonged (83, 84). The survival effect of bevacizumab on BC patients requires confirmation by more studies. Bevacizumab has been reported to cause heart failure, decreased cardiac function, and even stress cardiomyopathy—effects possibly related to factors such as reduced capillary density and endothelial dysfunction caused by the inhibition of VEGF pathways and increased cardiac afterload (85, 86).

### Antibody-drug conjugates (ADCs)

Recently developed ADCs are beneficial for the treatment of BC patients, including those with HER2-positive, triple-negative and some with HER2 low-expressing tumors (87). ADCs combine the advantages of highly targeted monoclonal antibodies and cytotoxic small molecules to reduce the systemic toxicity of off-target small-molecule cytotoxins and improve antitumor efficacy (88, 89). T-DM1 is the first groundbreaking ADC used in BC therapy, with an inherent advantage of a “magic bullet” and stable and significant clinical efficacy. The agent consists of trastuzumab, a non-reducing thioether junction and the microtubulin inhibitor methane derivative (DM1) (90). DM1 and vincristine have similar mechanisms of action, as they both inhibit microtubule protein polymerization by binding to them, which, in turn, induces cell cycle arrest and apoptosis. T-DM1 binding to HER2 allows the complex to enter target cells through receptor-mediated endocytosis. The antibody component of T-DM1 is degraded in the lysosomes, releasing DM1 into the cytoplasm, ultimately leading to cell cycle arrest and induction of cell death (91, 92). The EMILIA study, a pivotal phase III clinical study of T-DM1 for the second-line treatment of advanced HER2-positive BC, showed that T-DM1 significantly prolonged the median PFS and median OS with lower toxicity and better overall tolerability than lapatinib combined with capecitabine in HER2-positive advanced BC patients previously treated with paclitaxel and trastuzumab-based agents (60).

A recent study analyzing the occurrence of adverse drug reactions associated with HER2-positive BC through a spontaneous reporting system database showed that half of the adverse reactions associated with T-DM1 included blood disorders, such as thrombocytopenia, while cardiac disorders, including heart failure and arrhythmias, were significantly less frequent compared to their incidence associated with classical anti-HER2 antibodies (trastuzumab and pertuzumab), with an overall incidence of only 3% (93). Another study performed a pooled analysis of 1961 advanced HER2-positive BC cases treated with T-DM1 to determine the incidence of cardiotoxicity, its clinical presentation, and possible risk factors related to T-DM1-associated cardiotoxicity. The results of this study showed that cardiac events were uncommon in T-DM1-treated patients, with at least one cardiac event occurring in 3.37% of the entire study sample; the most common type of cardiac event was a low-level (grade 1 or 2) LVEF decline (~2.04%), and the majority (~79%) of patients who experienced a cardiac event achieved recovery of cardiac function at the end of the follow-up (within 1 year) (94). Patients treated with T-DM1 in the ATEMPT trial exhibited good prognoses and rarely experienced toxic effects, making T-DM1 a potential treatment option for selected stage I HER2-positive BC patients (95). Considering that cardiotoxicity is the most significant adverse event associated with trastuzumab (the main molecular component of T-DM1), researchers performed a subanalysis of the ATEMPT trial to determine the cardiac safety of adjuvant T-DM1 therapy. The results showed an incidence of grade 3–4 left ventricular systolic insufficiency of 0.8 and 1.8% in the T-DM1 and trastuzumab combined with paclitaxel groups, respectively. In addition, three patients (0.8%) in the T-DM1 group showed a significant asymptomatic decrease in LVEF compared with those in six patients (5.3%) in the paclitaxel combined with trastuzumab group. Follow-up data indicated that all patients achieved complete remission of any cardiac symptoms and their LVEF returned to normal levels (96).

A prospective multicenter phase III two-arm clinical trial enrolled 991 metastatic HER2-postive BC patients who received T-DM1 and capecitabine together with lapatinib in a 1:1 ratio. The incidence of LVEF <50% or at least a 15% reduction from baseline in the T-DM1 group was only 1.7%, and the incidence of class III left ventricular systolic dysfunction was only 0.2% (97). In the TH3RESA trial, patients who received at least two anti-HER2 agents (trastuzumab and lapatinib) and paclitaxel, had progressive disease, and no cardiovascular events were reported in patients who received T-DM1 rescue therapy (98).

### Poly (ADP-ribose) polymerase (PARP) inhibitors

Approximately 7–15% of HER2-negative BC patients carry BRCA 1/2 mutations. These patients are characterized by high aggressiveness, high recent/distant recurrence rate, and poor prognosis (99). PARP plays a crucial role in gene damage response, regulation of apoptosis, and maintenance of genomic stability (100). Cells with BRCA1 and BRCA2 mutations are exceptionally sensitive to PARP inhibitors; thus, PARP inhibitors are used to treat HR-deficient malignancies (101). The most commonly used commercially available PARP inhibitors include olaparib (AZD2281), niraparib (MK-4827), rucaparib, veliparib (ABT- 888), fluzoparib and talazoparib (BMN- 673) (102). Olaparib was the world's first commercially available PARP inhibitor. PARPase is closely related to DNA transcription and repair in humans and is an essential protein for cancer cell functionality, especially in patients with BRCA mutations. Notably, BRCA protein defects make tumor cells highly dependent on the PARPase action. Olaparib inhibits the activity of PARP, resulting in tumor cells apoptosis due to unresolved DNA damage (103). Olaparib shows a substantial clinical benefit in patients with advanced breast and ovarian cancers (104). Clinical safety data are lacking for olaparib as adjuvant treatment of BC patients. A recent Japanese study analyzed the adverse reactions to two PARP inhibitors, olaparib and niraparib, using the Japan Adverse Drug Reaction Reports database. In this study, 1,287 cases (83.0%) of adverse reactions caused by olaparib were reported in ovarian cancer patients, whereas 177 cases (11.4%) of adverse reactions were detected in BC patients. The most common adverse events with olaparib were hematologic reactions, gastrointestinal reactions, and general malaise, with common hematologic toxicities including anemia, decreased neutrophil count, and decreased platelet count. Mild and moderate olaparib-related adverse events may be related to both target and off-target effects of the drug, and most of such effects can be controlled by dose suspension, dose reduction and symptomatic treatment. Most adverse events occurred within 3 months of drug administration, after which the symptoms gradually resolved (105). Almost no cases of olaparib-induced cardiotoxicity have been reported.

### Programmed cell death protein 1 (PD-1) and programmed cell death 1 ligand 1 (PD-L1) monoclonal antibodies

Due to substantial research on the anti-tumoral properties of the immune system and on the immune characteristics of TNBC. ICIs represented by PD-1 and PD-L1 have become a viable therapeutic option for TNBC. In the tumor microenvironment, PD-L1 on the tumor cells surfaces combined with PD-1 on T cell surfaces can attenuate T cell-mediated immunosurveillance *via* various mechanisms, such as the induction of T cell non-response, failure, and even apoptosis; reducing the levels of cytokines such as tumor necrosis factor, interferon-γ, and interleukin-2; and inhibiting tumor infiltrating CD4-positive and CD8-positive T-cells (CD4^+^/CD8^+^ TILs), thus providing a way for cancer cells to evade the immune responses (106). High levels of PD-L1 and TILs are more often observed in TNBC than in other BC types, rendering TNBC more sensitive to immunotherapy (e.g., PD-1/PD-L1 inhibitors) (107). Studies have confirmed that blocking PD-1/PD-L1 activates and upregulates the expression of CD8^+^ T-cells, increases their tumor-killing activity, and induces the expression of major histocompatibility complex molecules on the surface of cancer cells, exposing more cancer cells to immune cells (108). A phase I clinical trial showed that atezolizumab treatment resulted in an ORR of 24% and a median OS of 17.6 months in TNBC patients, and that PD-L1-positive patients had a higher ORR (12% vs. 0) and longer OS (10.1 vs. 6.0 months) than PD-L1-negative patients (109). Phase II clinical trials have confirmed that pembrolizumab had an ORR of 5.3% and a disease control rate of 7.6% in TNBC patients without other treatments, with a median PFS and OS of 2 and 9 months, respectively (110). Durvalumab significantly improved the pCR (9.2%) in TNBC patients (111).

In clinical practice, the use of anti-PD-1 and PD-L1 agents has become increasingly common due to their effectiveness in prolonging the survival of patients with advanced disease. Consequently, immune-related adverse effects have been reported, including skin toxicity, endocrine toxicity, gastrointestinal toxicity, pulmonary toxicity, hepatotoxicity, nephrotoxicity, and cardiotoxicity. These side effects tend to be pronounced when immune modulators are used in combination with ICIs, and they require early detection and management (112). Cardiac complications have been reported in <1% of patients treated with ICIs and are considered rare and serious adverse events that are difficult to treat with glucocorticoids. The cardiotoxic effects include myocarditis, pericardial effusion, and arrhythmias, with autoimmune myocarditis being the most typical manifestation (113). In the treatment of BC, a case of a postoperative recurrent TNBC patient receiving chemotherapy combined with a PD-1 inhibitor resulting in severe immune-related hepatitis and myocarditis was reported. The patient's condition improved after methylprednisolone treatment, interruption of chemotherapy, and discontinuation of immunotherapy (114). The pathogenesis of myocarditis due to PD-1 inhibitors remains unclear, and autopsies of fatal cases presenting with myocarditis are often accompanied by massive inflammatory cell infiltration, increased extracellular space volume, and loss of cardiomyocytes. Analysis of these inflammatory-reactive cells has confirmed the presence of CD4^+^ and CD8^+^ T-cells in the myocardium (113). Palaskas et al., suggested that a possible mechanism of PD-1/PD-L1 ICI-induced myocarditis stems from the presence of two antigens shared between tumor cells and cardiomyocytes. One of them is a TCR targeting a different but homologous antigen to the muscle antigen that is the tumor antigen, and the other is a specific TCR targeting a different antigen (115). Therefore, the drug may attack the common antigen as a target, causing myocardial damage and the manifestation of myocarditis.

## Risk factors associated with cardiovascular events in targeted therapies

Recent guidelines published by the American Society of Clinical Oncology on the prevention and monitoring of cardiovascular events in adult oncology survivors (116) stated that patients who have received prior anthracycline-based chemotherapy regimens, particularly those who received cumulative doses of doxorubicin over 250 mg/m^2^ or epirubicin over 600 mg/m^2^, were at high risk of cardiac insufficiency at follow-up and may experience later cardiovascular events. A retrospective analysis suggested that prior anthracycline use was the only risk factor that was significantly associated with trastuzumab-related cardiotoxicity (117). The concomitant use of anthracycline with trastuzumab or sequential trastuzumab therapy too close to the previous chemotherapy treatment with anthracycline (3 weeks vs. 3 months) may increase the incidence of adverse cardiac events (118). In the N9831 trial (119), trastuzumab-related cardiotoxicity was more common in patients aged ≥60 years with low baseline LVEF levels and on antihypertensive medications. Likewise, long-term follow-up results from the NSABP-B31 trial showed that higher age and a low baseline LVEF of 50% to 54% were significantly associated with adverse cardiovascular events induced by trastuzumab treatment (120). Trastuzumab treatment exacerbated the deterioration of cardiac function in older patients, and the incidence of cardiovascular events increased significantly with age (118). In patients receiving trastuzumab-T-DM1 as adjuvant therapy, age (>65 years) was the most important clinical factor for the occurrence of cardiac events, and advanced age can increase the risk of cardiac events by at least 5%. These data indicate that older age is a risk factor for trastuzumab-related cardiotoxicity (121). A meta-analysis suggested that a higher body mass index (BMI >25 or >30 kg/m^2^) was significantly associated with the occurrence of trastuzumab-related cardiotoxicity, with cardiovascular event rates 1.32 times and 1.47 times higher than those of patients with a normal BMI, respectively (122). Due to the lack of data from a large prospective phase III trial, it is unclear whether the incidence of severe adverse cardiac events during trastuzumab treatment is significantly higher in patients with a combined history of prior cardiovascular disease and impaired LVEF at baseline and whether this affects overall patient survival.

## Monitoring of cardiovascular toxicity associated with targeted BC therapy

Echocardiography, ECG, cardiac magnetic resonance, and cardiac serum biomarkers are commonly used in clinical monitoring. Echocardiography is the preferred method for monitoring cardiac impairment before and after treatment in cancer patients, given the method's non-invasiveness, universality, and convenience of use. The widely used monitoring index is LVEF, but LVEF characterized by low sensitivity to early cardiac impairment (which can be easily overlooked), resulting in patients missing the most optimal treatment period. In recent years, the GLS based on two-dimensional speckle tracking technology has been measured with low error, and GLS decreases earlier than LVEF in echocardiography of cancer patients; therefore, monitoring GLS during treatment can detect early cardiovascular toxicity (123). ECG has the advantages of being easy to perform, noninvasive, and highly reproducible. If an abnormal ECG which refers to the occurrence of pathological Q wave, ST segment elevation, QRS wave distortion, ruptured QRS wave, QT interval extension or shortening and P wave deviation, is found during targeted therapy, it should be combined with clinical administration of protective and nutritive myocardial drugs or drug replacement. ECG changes in BC patients after targeted therapy are non-specific and may be due to other reasons. However, abnormal ECG changes after drug administration with normal ECG before treatment can be combined with clinical findings, suggesting the possibility of treatment-related cardiovascular toxicity. Currently, traditional predictors of cardiovascular toxicity such as cardiac troponin (cTn) and amino-terminal pro-B-type natriuretic peptide (NT-proBNP), are commonly used in oncology clinical practice (124). When cardiomyocytes are damaged, cTnT and cTnI are rapidly released into the blood, and their plasma concentrations can reflect the cardiovascular toxicity of early antitumor drugs (125, 126). Baseline measurements of BNP/NT-proBNP and cTn T/I should be performed prior to oncologic therapy to provide baseline values. Because baseline values may be elevated when other cardiovascular diseases are present, they are particularly important for accurate interpretation of changes in subsequent serum markers during monitoring or changes in serum markers when new cardiovascular symptoms develop. Changes in cardiac biomarkers should be monitored regularly during and after tumor treatment for early detection of myocardial injury. CMR imaging is the gold standard for measuring left ventricular volumes and left ventricular systolic function in noninvasive examinations, and it has the advantage of being accurate and reproducible in measuring ventricular volumes. CMR imaging also detects inflammatory changes during early myocardial injury, edema, and advanced myocardial fibrosis. It has great advantages in evaluating cardiac function and myocardial histology (127). Endomyocardial biopsy (EMB) is the most sensitive and specific method for the assessment of cardiovascular toxicity caused by antineoplastic drugs. Based on the extent of EMB tissue and cell involvement, clinicians can grade and assess cardiovascular toxicity, but noninvasive tests cannot replace EMB, which remains the gold standard for confirming myocarditis, inflammatory cardiomyopathy, and infiltrative heart disease (128). However, the application of EMB is limited due to its invasiveness.

According to the 2022 ESC Guidelines for Oncological Cardiology, monitoring modalities during antineoplastic therapy should include 3D echocardiography, GLS, and cardiac biomarkers to detect the risk of cardiovascular toxicity based on specific antineoplastic therapy (27). With anti-VEGF monoclonal antibodies, for intermediate-risk or high-risk patients with prolonged QTc interval, monthly electrocardiograms are recommended 3 months after treatment initiation and every 3–6 months thereafter. For high-risk patients, electrocardiograms are performed 2 weeks after the start of treatment, and new assessments are performed at any dose increase. Studies have shown that anti-HER2 therapy may lead to LVD in up to 15–20% of patients, resulting in severe heart failure if monitoring is missed, or in high-risk and very high-risk patients. Therefore, early monitoring is of crucial importance in these patients. Ideally, the evaluation should be conducted before initiating trastuzumab treatment. Cardiac color Doppler ultrasound should be performed every 3 weeks during the second or third week of the trastuzumab therapy cycle. For low-risk HER2+ early BC patients who have no clinical symptoms after 3 months and have normal color Doppler echocardiography findings, monitoring can be reduced to once every 4 months. The guidelines also recommend that all patients undergo ECG, NP, and cTn monitoring prior to ICI therapy. Baseline echocardiography is recommended for high-risk patients before ICI therapy initiation. ECG and cTn should be monitored continuously until the 2nd, 3rd, and 4th doses of ICI, and if normal, reduced to testing every 3rd dose until treatment completion. For patients requiring long-term (i.e., >12 months) therapy, CV evaluation can be considered every 6–12 months, depending on the specific situation (especially in high-risk patients).

The following are specific monitoring recommendations for targeted therapy. (1) ECG: a. routine ECG should be performed before each treatment cycle; b. ECG should be performed at any time if the patient develops relevant clinical symptoms, signs, or relevant index abnormalities. (2) Echocardiography (GLS, LVEF): a. Baseline screening should be initiated before tumor treatment; b. Echocardiography should be reviewed upon every two treatment cycles for high-risk patients and every two to four treatment cycles for low- and medium-risk patients; c. Echocardiography should be performed whenever symptoms, signs, or risk factors of myocardial injury appear during treatment, and the frequency of monitoring afterwards should be determined according to the patient's condition; d. Echocardiography should be performed 6–12 months after the end of treatment and should be repeated periodically thereafter. (3) Biomarkers (cTn, BNP, NT-proBNP): a. All patients should be screened for biomarkers prior to cancer therapy; b. Biomarkers should be reviewed every one to two treatment cycles for high-risk patients and every two to four treatment cycles for low- and intermediate-risk patients (for HER2-targeted therapy, biomarkers should be monitored before and after each treatment cycle for the first 3–6 months). c. Biomarker testing should be performed at any time when symptoms, signs, or risk factors of myocardial injury appear during treatment, and the frequency of monitoring should be determined based on the patient's condition. (4) Radiography, CMR imaging, EMB: not routinely recommended, depending on the specific condition and clinical needs. The risk stratification of cardiovascular toxicity related to targeted drugs for breast cancer is shown in Table 2.

**Table 2:** Risk stratification of cardiovascular toxicity associated with targeted drugs for breast cancer.

	**Treatment related risk factors**	**Patient related risk factors**
Low risk	No anthracycline drugs before application of trastuzumab	Age: >18 and <50
Medium risk	Application of trastuzumab, VEGF, tyrosine kinase inhibitor after anthracycline drugs	Age: 50-64 years old; 1–2 cardiovascular disease risk factors, such as hypertension, diabetes/insulin resistance, dyslipidemia, smoking, obesity
High risk	Simultaneous use of anthracyclines and trastuzumab; Patients who have received anthracycline chemotherapy should be treated with VEGF and tyrosine kinase inhibitors	Age ≥ 65 years old; Combined with more than 2 cardiovascular disease risk factors, such as hypertension, diabetes/insulin resistance, dyslipidemia, smoking, obesity; Complicated with cardiovascular diseases, such as coronary heart disease, peripheral vascular disease, cardiomyopathy, serious valvular heart disease, heart failure, arrhythmia (atrial fibrillation, atrial flutter, ventricular tachycardia, etc.); LVEF has decreased before receiving tumor treatment, or LVEF is close to the lower limit of normal value (LVEF 50–54%)

## Interventions for targeted therapy-associated cardiotoxicity

### Interruption of targeted therapy

The greatest consequence of trastuzumab-related cardiotoxicity is interruption of targeted therapy. Since most patients have been treated with anthracyclines, they have a significantly higher rate of cardiovascular events during trastuzumab therapy. However, the interruption of targeted therapy may be associated with an increased rate of tumor recurrence (129). Among HER2-postive BC patients treated with trastuzumab, 13.5% are forced to discontinue treatment due to associated cardiovascular events (30% due to heart failure and 70% due to asymptomatic LVEF decline). In most trastuzumab treatment trials, the treatment was discontinued when patients exhibited signs of chronic congestive heart failure or had LVEF below 45% (130). Currently, trastuzumab therapy should be suspended when LVEF decreases by ≥15% in absolute terms from pretreatment baseline levels, or falls below the normal range and decreases by ≥10% in absolute terms from pretreatment baseline levels, or when clinical manifestations of chronic congestive heart failure are present. If LVEF returns to the normal range or decreases ≤ 10% in absolute terms from pretreatment levels within 4–8 weeks, trastuzumab therapy may be resumed. Trastuzumab should be permanently discontinued if LVEF continues to decline for >8 weeks or if more than three discontinuations are implemented due to cardiovascular events (118).

### Pharmacologic interventions

Several observational studies and small randomized clinical trials suggest that early use of angiotensin-converting enzyme inhibitors (ACEI) and β-blockers in the presence of cardiovascular events during anthracycline and trastuzumab therapy may improve cardiac event outcomes (118). A small prospective study further demonstrated that the combination of ACEI/angiotensin receptor blockers (ARB) with beta-blockers consistently improved cardiac function between months 3 and 12 of trastuzumab treatment and significantly increased the probability that LVEF would return to normal by the end of trastuzumab therapy (131). The PRADA trial aimed to test the hypothesis that the concomitant administration of an ACEI/ARB, a β-blocker, or a combination of both, during adjuvant anthracycline chemotherapy and trastuzumab treatment would reduce the incidence of cardiotoxicity (132). The trial included 130 patients who were postoperatively prepared to receive the FEC ± sequential trastuzumab adjuvant regimen and were randomized to the candesartan 32 mg qd + metoprolol 100 mg qd group, the candesartan 32 mg qd + placebo group, the metoprolol 100 mg qd + placebo group, or the placebo + placebo group according to a balanced 1:1:1:1 rationing pattern. The primary endpoint event was defined as a 5% change in LVEF from baseline levels as determined *via* cardiac MRI measurements and was considered clinically significant. The results of the PRADA trial showed that concomitant candesartan treatment in early-stage HER2-postive BC patients treated with anthracycline-based adjuvant chemotherapy with sequential trastuzumab was effective in reversing the early decline in LVEF. However, a recent clinical trial of concomitant candesartan during trastuzumab treatment yielded different results than the those of the PRADA trial (133). In another small-sample randomized controlled trial, patients were randomized to receive bisoprolol, perindopril, or placebo during adjuvant trastuzumab treatment, and at the end of targeted therapy, LV end-diastolic volume and LVEF were remeasured (134). No significant difference was observed in the increase in LV end-diastolic volume from baseline among the three groups, but a statistically significant difference was identified in the change in LVEF from baseline, suggesting that bisoprolol and perindopril may slightly reduce the incidence of cardiovascular events during trastuzumab treatment, without significantly improving cardiac outcomes. Based on this limited evidence, the American Heart Association (AHA) recommends that patients receiving trastuzumab be started on an ACEI or ARB after any clinical evidence of significant cardiac impairment has been identified. The evidence of significant abnormalities in cardiac function includes: (1) a decrease in LVEF >15% or LVEF <50% without clinical signs of cardiac insufficiency and (2) changes in GLS of more than 15% (130).

### Non-pharmacological therapies

Exercise is an effective multi-targeted therapy to prevent and treat multiple competing mechanisms of cancer therapy-related cardiovascular toxicity in cancer survivors, including cardiorespiratory fitness (CRF) impairment, cardiovascular injury, and pre-existing and new cardiovascular risk factors. Several long-term follow-up studies and meta-analyses of BC patients have shown that increasing physical activity boosts maximal oxygen uptake and significantly reduces cardiovascular events and CVD mortality (135–137), and the AHA Statement on Cardiac Rehabilitation for Oncology Cardiac Patients suggests that exercise training is feasible and effective in cancer survivors, improving their CRF, muscle strength, and quality of life (138). Dolan et al., reported that among 152 BC survivors, patients in a cardiac rehabilitation (CR) group who underwent aerobic and resistance training once a week postoperatively showed significant improvements in CRF, quality of life, and fatigue at a mean follow-up of (177 ± 167) weeks postoperatively (139). CR, an important component of the secondary prevention of CVD, is relatively new concept in oncology, and more studies are needed to confirm the therapeutic efficacy of CR in reducing cardiotoxicity associated with neoadjuvant targeted therapy in BC.

### Interventional strategies for cardiotoxicity due to novel targeted drugs

Novel anti-HER2 drugs (such as pertuzumab), and rTKI (represented by lapatinib) have the same cardiotoxic effects as trastuzumab; however, novel anti-HER-2 drugs (e.g., lapatinib) exert off-target cardioprotective effects in basic studies and generally cause a lower incidence of cardiovascular events than trastuzumab during neoadjuvant, late first-line, or rescue therapy (26, 41, 140). Since most patients have already received trastuzumab or its combination regimens, interventions for cardiovascular events due to novel anti-HER2 drugs are mainly based on primary and secondary preventive measures for trastuzumab-related cardiotoxicity while administering individualized treatment.

## Conclusion and future perspectives

Targeted therapeutic drugs for BC, including trastuzumab, patuzumab, lapatinib, rebosinib, and bevacizumab are associated with cardiovascular toxicity. The main manifestations of the latter include LVEF decline, left ventricular dysfunction, heart failure, cardiac death, hypertension and other rare side effects. The mechanisms of cardiovascular injury induced by targeted drugs are diverse, including direct effects on the heart or vascular system, the release of cardiac regulators, and alterations in coagulation status. Therefore, cardiac function should be monitored regularly during targeted therapy. With the growing use of targeted drugs, the associated cardiovascular toxicity is being increasingly recognized, and multiple cardiovascular toxicity detection and treatment methods are being developed and tested.

An ongoing randomized, open-label, phase II pilot study in BC patients receiving neoadjuvant therapy aims to determine whether the co-administration of metformin and doxorubicin reduces the number of patients who develop a significant change in LVEF (NCT02472353). Another registry study aimed to analyze the protective impact of beta-blockers and ACEIs for BC patients treated with anthracycline-based chemotherapy with or without trastuzumab using myocardial strain imaging monitoring is active (NCT02236806). The readouts of these studies will provide novel insights into our understanding of treatment for cardiac toxicity caused by neoadjuvant targeted therapy in BC patients.

Current preventive and treatment strategies for cardiotoxicity associated with anti-HER2 therapy include detailed cardiovascular evaluation prior to treatment and avoidance of concomitant use of drugs with cardiovascular toxicity (e.g., anthracyclines). If both measures are implemented together, closer monitoring of cardiovascular function is required, and early intervention for cardiac decompensation during treatment (e.g., decreased LVEF ejection function) is necessary. Once severe cardiac decompensation occurs during treatment, anti-HER2 therapy must be discontinued, and this patient group faces a higher risk of tumor recurrence and additional cardiovascular disease burden, which severely affects the prognosis. Therefore, future research should focus on the cardiovascular safety of patients with impaired baseline cardiac function receiving targeted therapy, with emphasis on the development of more sensitive cardiac function assays and more aggressive cardiovascular interventions during cancer treatment.

## Author contributions

Conceptualization: YL and YY. Writing—original draft preparation: YL. Writing—review and editing: LZ, XZ, and YY. Supervision: XZ and YY. Validation: XC and YY. Funding acquisition: XZ. All authors have read and agreed to the published version of the manuscript.

## References

[1] Sung H Ferlay J Siegel RL Laversanne M Soerjomataram I Jemal A. Global Cancer Statistics 2020: GLOBOCAN Estimates of Incidence and Mortality Worldwide for 36 Cancers in 185 Countries. CA Cancer J Clin. (2021) 71:209–49. 10.3322/caac.21660 33538338

[2] Mcdonald ES Clark AS Tchou J Zhang P Freedman GM. Clinical Diagnosis and Management of Breast Cancer. J Nucl Med. (2016) 57:9S–16S. 10.2967/jnumed.115.157834 26834110

[3] The L. Breast cancer targeted therapy: successes and challenges. Lancet. (2017) 389:2350. 10.1016/S0140-6736(17)31662-8 28635596

[4] Gradishar WJ Moran MS Abraham J Aft R Agnese D Allison KH. Breast Cancer, Version 3.2022, NCCN Clinical Practice Guidelines in Oncology. J Natl Compr Canc Netw. (2022) 20:691–722. 35714673 10.6004/jnccn.2022.0030

[5] Spring LM Bar Y Isakoff SJ. The Evolving Role of Neoadjuvant Therapy for Operable Breast Cancer. J Natl Compr Canc Netw. (2022) 20:723–34. 10.6004/jnccn.2022.7016 35714678

[6] Korde LA Somerfield MR Carey LA Crews JR Denduluri N Hwang ES. Neoadjuvant Chemotherapy, Endocrine Therapy, and Targeted Therapy for Breast Cancer: ASCO Guideline. J Clin Oncol. (2021) 39:1485–505. 10.1200/JCO.20.03399 33507815 PMC8274745

[7] Wuerstlein R Harbeck N. Neoadjuvant Therapy for HER2-positive Breast Cancer. Rev Recent Clin Trials. (2017) 12:81–92. 10.2174/1574887112666170202165049 28164759

[8] Harbeck N. Neoadjuvant and adjuvant treatment of patients with HER2-positive early breast cancer. Breast. (2022) 62:S12–S6. 10.1016/j.breast.2022.01.006 35148934 PMC9097807

[9] Huang M O'shaughnessy J Zhao J Haiderali A Cortes J Ramsey SD. Association of pathologic complete response with long-term survival outcomes in triple-negative breast cancer: a meta-analysis. Cancer Res. (2020) 80:5427–34. 10.1158/0008-5472.CAN-20-1792 32928917

[10] Gao M Fu C Li S Chen F Yang Y Wang C. The efficacy and safety of pyrotinib in treating HER2-positive breast cancer patients with brain metastasis: A multicenter study. Cancer Med. (2022) 11:735–42. 10.1002/cam4.4481 34962098 PMC8817079

[11] Krop I Ismaila N Andre F Bast RC Barlow W Collyar DE. Use of biomarkers to guide decisions on adjuvant systemic therapy for women with early-stage invasive breast cancer: american society of clinical oncology clinical practice guideline focused update. J Clin Oncol. (2017) 35:2838–47. 10.1200/JCO.2017.74.0472 28692382 PMC5846188

[12] Seshadri R Firgaira FA Horsfall DJ Mccaul K Setlur V Kitchen P. Clinical significance of HER-2/neu oncogene amplification in primary breast cancer. The South Australian Breast Cancer Study Group. J Clin Oncol. (1993) 11:1936–42. 10.1200/JCO.1993.11.10.1936 8105035

[13] Press MF Pike MC Chazin VR Hung G Udove JA Markowicz M. Her-2/neu expression in node-negative breast cancer: direct tissue quantitation by computerized image analysis and association of overexpression with increased risk of recurrent disease. Cancer Res. (1993) 53:4960–70. doi:8104689

[14] Saad ED Squifflet P Burzykowski T Quinaux E Delaloge S Mavroudis D. Disease-free survival as a surrogate for overall survival in patients with HER2-positive, early breast cancer in trials of adjuvant trastuzumab for up to 1 year: a systematic review and meta-analysis. Lancet Oncol. (2019) 20:361–70. 10.1016/S1470-2045(18)30750-2 30709633 PMC7050571

[15] Davey MG Browne F Miller N Lowery AJ Kerin MJ. Pathological complete response as a surrogate to improved survival in human epidermal growth factor receptor-2-positive breast cancer: systematic review and meta-analysis. BJS Open. (2022) 6:zrac028. 10.1093/bjsopen/zrac028 35512244 PMC9071230

[16] Saura C Oliveira M Feng YH Dai MS Chen SW Hurvitz SA. Neratinib Plus Capecitabine Versus Lapatinib Plus Capecitabine in HER2-Positive Metastatic Breast Cancer Previously Treated With >/= 2 HER2-Directed Regimens: Phase III NALA Trial. J Clin Oncol. (2020) 38:3138–49. 10.1200/JCO.20.00147 32678716 PMC7499616

[17] Grassi L Biancosino B Marmai L Righi R. Effect of reboxetine on major depressive disorder in breast cancer patients: an open-label study. J Clin Psychiatry. (2004) 65:515–20. 10.4088/JCP.v65n0410 15119914

[18] Koutras AK Fountzilas G Makatsoris T Peroukides S Kalofonos HP. Bevacizumab in the treatment of breast cancer. Cancer Treat Rev. (2010) 36:75–82. 10.1016/j.ctrv.2009.10.007 19932567

[19] Marks DK Kalinsky K. Bevacizumab in breast cancer: a targeted therapy still in search of a target population. Semin Oncol. (2017) 44:286–7. 10.1053/j.seminoncol.2017.10.011 29526257

[20] Zhao YY Sawyer DR Baliga RR Opel DJ Han X Marchionni MA. Neuregulins promote survival and growth of cardiac myocytes. Persistence of ErbB2 and ErbB4 expression in neonatal and adult ventricular myocytes. J Biol Chem. (1998) 273:10261–9. 10.1074/jbc.273.17.10261 9553078

[21] Yang Z Wang W Wang X Qin Z. Cardiotoxicity of Epidermal Growth Factor Receptor 2-Targeted Drugs for Breast Cancer. Front Pharmacol. (2021) 12:741451. 10.3389/fphar.2021.741451 34790121 PMC8591078

[22] Lunardi M Al-Habbaa A Abdelshafy M Davey MG Elkoumy A Ganly S. Genetic and RNA-related molecular markers of trastuzumab-chemotherapy-associated cardiotoxicity in HER2 positive breast cancer: a systematic review. BMC Cancer. (2022) 22:396. 10.1186/s12885-022-09437-z 35413811 PMC9004047

[23] Smith AE Ferraro E Safonov A Morales CB Lahuerta EJA Li Q. HER2 + breast cancers evade anti-HER2 therapy via a switch in driver pathway. Nat Commun. (2021) 12:6667. 10.1038/s41467-021-27093-y 34795269 PMC8602441

[24] Lee J Liu H Pearson T Iwase T Fuson J Lalani AS. PI3K and MAPK Pathways as Targets for Combination with the Pan-HER Irreversible Inhibitor Neratinib in HER2-Positive Breast Cancer and TNBC by Kinome RNAi Screening. Biomedicines. (2021) 9:740. 10.3390/biomedicines9070740 34203351 PMC8301343

[25] Curigliano G Viale G Bagnardi V Fumagalli L Locatelli M Rotmensz N. Clinical relevance of HER2 overexpression/amplification in patients with small tumor size and node-negative breast cancer. J Clin Oncol. (2009) 27:5693–9. 10.1200/JCO.2009.22.0962 19884553

[26] Albini A Cesana E Donatelli F Cammarota R Bucci EO Baravelli M. Cardio-oncology in targeting the HER receptor family: the puzzle of different cardiotoxicities of HER2 inhibitors. Future Cardiol. (2011) 7:693–704. 10.2217/fca.11.54 21929348

[27] Lyon AR Lopez-Fernandez T Couch LS Asteggiano R Aznar MC Bergler-Klein J. 2022 ESC Guidelines on cardio-oncology developed in collaboration with the European Hematology Association (EHA), the European Society for Therapeutic Radiology and Oncology (ESTRO) and the International Cardio-Oncology Society (IC-OS). Eur Heart J. (2022) 43:4229–361. 10.1093/eurheartj/ehac244 36017568

[28] Mamtani A Barrio AV King TA Van Zee KJ Plitas G Pilewskie M. How often does neoadjuvant chemotherapy avoid axillary dissection in patients with histologically confirmed nodal metastases? Results of a Prospective Study. Ann Surg Oncol. (2016) 23:3467–74. 10.1245/s10434-016-5246-8 27160528 PMC5070651

[29] Burstein HJ Curigliano G Thurlimann B Weber WP Poortmans P Regan MM. Customizing local and systemic therapies for women with early breast cancer: the St. Gallen International Consensus Guidelines for treatment of early breast cancer 2021. Ann Oncol. (2021) 32:1216–35. 10.1016/j.annonc.2021.06.023 34242744 PMC9906308

[30] Cortazar P Zhang L Untch M Mehta K Costantino JP Wolmark N. Pathological complete response and long-term clinical benefit in breast cancer: the CTNeoBC pooled analysis. Lancet. (2014) 384:164–72. 10.1016/S0140-6736(13)62422-8 24529560

[31] Von Minckwitz G Huang CS Mano MS Loibl S Mamounas EP Untch M. Trastuzumab emtansine for residual invasive HER2-positive breast cancer. N Engl J Med. (2019) 380:617–28. 10.1056/NEJMoa1814017 30516102

[32] Chan A Delaloge S Holmes FA Moy B Iwata H Harvey VJ. Neratinib after trastuzumab-based adjuvant therapy in patients with HER2-positive breast cancer (ExteNET): a multicentre, randomised, double-blind, placebo-controlled, phase 3 trial. Lancet Oncol. (2016) 17:367–77. 10.1016/S1470-2045(15)00551-3 26874901

[33] Cameron D Piccart-Gebhart MJ Gelber RD Procter M Goldhirsch A De Azambuja E. 11 years' follow-up of trastuzumab after adjuvant chemotherapy in HER2-positive early breast cancer: final analysis of the HERceptin Adjuvant (HERA) trial. Lancet. (2017) 389:1195–205. 10.1016/S0140-6736(16)32616-2 28215665 PMC5465633

[34] An J Sheikh MS. Toxicology of trastuzumab: an insight into mechanisms of cardiotoxicity. Curr Cancer Drug Targets. (2019) 19:400–7. 10.2174/1568009618666171129222159 29189161

[35] Brandao SR Carvalho F Amado F Ferreira R Costa VM. Insights on the molecular targets of cardiotoxicity induced by anticancer drugs: A systematic review based on proteomic findings. Metabolism. (2022) 134:155250. 10.1016/j.metabol.2022.155250 35809654

[36] Maximiano S Magalhaes P Guerreiro MP Morgado M. Trastuzumab in the treatment of breast cancer. BioDrugs. (2016) 30:75–86. 10.1007/s40259-016-0162-9 26892619

[37] Piccart M Procter M Fumagalli D De Azambuja E Clark E Ewer MS. Adjuvant pertuzumab and trastuzumab in early HER2-positive breast cancer in the APHINITY trial: 6 years' follow-up. J Clin Oncol. (2021) 39:1448–57. 10.1200/JCO.20.01204 33539215

[38] Goutsouliak K Veeraraghavan J Sethunath V De Angelis C Osborne CK Rimawi MF. Towards personalized treatment for early stage HER2-positive breast cancer. Nat Rev Clin Oncol. (2020) 17:233–50. 10.1038/s41571-019-0299-9 31836877 PMC8023395

[39] Gianni L Pienkowski T Im YH Roman L Tseng LM Liu MC. Efficacy and safety of neoadjuvant pertuzumab and trastuzumab in women with locally advanced, inflammatory, or early HER2-positive breast cancer (NeoSphere): a randomised multicentre, open-label, phase 2 trial. Lancet Oncol. (2012) 13:25–32. 10.1016/S1470-2045(11)70336-9 22153890

[40] Nami B Maadi H Wang Z. Mechanisms Underlying the Action and Synergism of Trastuzumab and Pertuzumab in Targeting HER2-Positive Breast Cancer. Cancers (Basel). (2018) 10:342. 10.3390/cancers10100342 30241301 PMC6210751

[41] Sendur MA Aksoy S Altundag K. Cardiotoxicity of novel HER2-targeted therapies. Curr Med Res Opin. (2013) 29:1015–24. 10.1185/03007995.2013.807232 23692263

[42] Slamon DJ Leyland-Jones B Shak S Fuchs H Paton V Bajamonde A. Use of chemotherapy plus a monoclonal antibody against HER2 for metastatic breast cancer that overexpresses HER2. N Engl J Med. (2001) 344:783–92. 10.1056/NEJM200103153441101 11248153

[43] Herrmann J. Adverse cardiac effects of cancer therapies: cardiotoxicity and arrhythmia. Nat Rev Cardiol. (2020) 17:474–502. 10.1038/s41569-020-0348-1 32231332 PMC8782611

[44] Canale ML Camerini A Huqi A Lilli A Bisceglia I Parrini I. Cardiovascular risk factors and timing of anthracyclines and trastuzumab cardiac toxicity. Anticancer Res. (2019) 39:5741–5. 10.21873/anticanres.13775 31570476

[45] Gianni L Pienkowski T Im YH Tseng LM Liu MC Lluch A. 5-year analysis of neoadjuvant pertuzumab and trastuzumab in patients with locally advanced, inflammatory, or early-stage HER2-positive breast cancer (NeoSphere): a multicentre, open-label, phase 2 randomised trial. Lancet Oncol. (2016) 17:791–800. 10.1016/S1470-2045(16)00163-7 27179402

[46] Swain SM Kim SB Cortes J Ro J Semiglazov V Campone M. Pertuzumab, trastuzumab, and docetaxel for HER2-positive metastatic breast cancer (CLEOPATRA study): overall survival results from a randomised, double-blind, placebo-controlled, phase 3 study. Lancet Oncol. (2013) 14:461–71. 10.1016/S1470-2045(13)70130-X 23602601 PMC4076842

[47] Swain SM Miles D Kim SB Im YH Im SA Semiglazov V. Pertuzumab, trastuzumab, and docetaxel for HER2-positive metastatic breast cancer (CLEOPATRA): end-of-study results from a double-blind, randomised, placebo-controlled, phase 3 study. Lancet Oncol. (2020) 21:519–30. 10.1016/S1470-2045(19)30863-0 32171426

[48] Denegri A Moccetti T Moccetti M Spallarossa P Brunelli C Ameri P. Cardiac toxicity of trastuzumab in elderly patients with breast cancer. J Geriatr Cardiol. (2016) 13:355–63. 10.11909/j.issn.1671-5411.2016.04.003 27403145 PMC4921548

[49] Giordano G Spagnuolo A Olivieri N Corbo C Campagna A Spagnoletti I. Cancer drug related cardiotoxicity during breast cancer treatment. Expert Opin Drug Saf. (2016) 15:1063–74. 10.1080/14740338.2016.1182493 27120499

[50] Henri C Heinonen T Tardif JC. The role of biomarkers in decreasing risk of cardiac toxicity after cancer therapy. Biomark Cancer. (2016) 8:39–45. 10.4137/BIC.S31798 27257396 PMC4878717

[51] Sandoo A Kitas GD Carmichael AR. Breast cancer therapy and cardiovascular risk: focus on trastuzumab. Vasc Health Risk Manag. (2015) 11:223–8. 10.2147/VHRM.S69641 25897242 PMC4397929

[52] Padegimas A Clasen S Ky B. Cardioprotective strategies to prevent breast cancer therapy-induced cardiotoxicity. Trends Cardiovasc Med. (2020) 30:22–8. 10.1016/j.tcm.2019.01.006 30745071 PMC7287268

[53] Varga ZV Ferdinandy P Liaudet L Pacher P. Drug-induced mitochondrial dysfunction and cardiotoxicity. Am J Physiol Heart Circ Physiol. (2015) 309:H1453–67. 10.1152/ajpheart.00554.2015 26386112 PMC4666974

[54] Varricchi G Ameri P Cadeddu C Ghigo A Madonna R Marone G. Antineoplastic drug-induced cardiotoxicity: a redox perspective. Front Physiol. (2018) 9:167. 10.3389/fphys.2018.00167 29563880 PMC5846016

[55] Rocca C De Francesco EM Pasqua T Granieri MC De Bartolo A Gallo Cantafio ME. Mitochondrial determinants of anti-cancer drug-induced cardiotoxicity. Biomedicines. (2022) 10:520. 10.3390/biomedicines10030520 35327322 PMC8945454

[56] De Azambuja E Procter MJ Van Veldhuisen DJ Agbor-Tarh D Metzger-Filho O Steinseifer J. Trastuzumab-associated cardiac events at 8 years of median follow-up in the Herceptin Adjuvant trial (BIG 1-01). J Clin Oncol. (2014) 32:2159–65. 10.1200/JCO.2013.53.9288 24912899

[57] Telli ML Hunt SA Carlson RW Guardino AE. Trastuzumab-related cardiotoxicity: calling into question the concept of reversibility. J Clin Oncol. (2007) 25:3525–33. 10.1200/JCO.2007.11.0106 17687157

[58] Cameron D Casey M Press M Lindquist D Pienkowski T Romieu CG. A phase III randomized comparison of lapatinib plus capecitabine versus capecitabine alone in women with advanced breast cancer that has progressed on trastuzumab: updated efficacy and biomarker analyses. Breast Cancer Res Treat. (2008) 112:533–43. 10.1007/s10549-007-9885-0 18188694

[59] Spector NL Xia W Burris H. 3rd, Hurwitz H, Dees EC, Dowlati A, et al. Study of the biologic effects of lapatinib, a reversible inhibitor of ErbB1 and ErbB2 tyrosine kinases, on tumor growth and survival pathways in patients with advanced malignancies. J Clin Oncol. (2005) 23:2502–12. 10.1200/JCO.2005.12.157 15684311

[60] Dieras V Miles D Verma S Pegram M Welslau M Baselga J. Trastuzumab emtansine versus capecitabine plus lapatinib in patients with previously treated HER2-positive advanced breast cancer (EMILIA): a descriptive analysis of final overall survival results from a randomised, open-label, phase 3 trial. Lancet Oncol. (2017) 18:732–42. 10.1016/S1470-2045(17)30312-1 28526536 PMC5531181

[61] Jerusalem G Lancellotti P Kim SB. HER2+ breast cancer treatment and cardiotoxicity: monitoring and management. Breast Cancer Res Treat. (2019) 177:237–50. 10.1007/s10549-019-05303-y 31165940 PMC6661020

[62] Blackwell KL Burstein HJ Storniolo AM Rugo H Sledge G Koehler M. Randomized study of Lapatinib alone or in combination with trastuzumab in women with ErbB2-positive, trastuzumab-refractory metastatic breast cancer. J Clin Oncol. (2010) 28:1124–30. 10.1200/JCO.2008.21.4437 20124187

[63] Geyer CE Forster J Lindquist D Chan S Romieu CG Pienkowski T. Lapatinib plus capecitabine for HER2-positive advanced breast cancer. N Engl J Med. (2006) 355:2733–43. 10.1056/NEJMoa064320 17192538

[64] Scott LJ. Apatinib: a review in advanced gastric cancer and other advanced cancers. Drugs. (2018) 78:747–58. 10.1007/s40265-018-0903-9 29663291

[65] Liu J Liu Q Li Y Li Q Su F Yao H. Efficacy and safety of camrelizumab combined with apatinib in advanced triple-negative breast cancer: an open-label phase II trial. J Immunother Cancer. (2020) 8:e000696. 10.1136/jitc-2020-000696 32448804 PMC7252975

[66] Fan M Zhang J Wang Z Wang B Zhang Q Zheng C. Phosphorylated VEGFR2 and hypertension: potential biomarkers to indicate VEGF-dependency of advanced breast cancer in anti-angiogenic therapy. Breast Cancer Res Treat. (2014) 143:141–51. 10.1007/s10549-013-2793-6 24292957

[67] Liu Z Shan J Yu Q Wang X Song X Wang F. Real-world data on apatinib efficacy—results of a retrospective study in metastatic breast cancer patients pretreated with multiline treatment. Front Oncol. (2021) 11:643654. 10.3389/fonc.2021.643654 34178630 PMC8224527

[68] Wang W He Q Li C Zhuang C Zhang H Wang Q. Research on the mechanism and prevention of hypertension caused by apatinib through the RhoA/ROCK signaling pathway in a mouse model of gastric cancer. Front Cardiovasc Med. (2022) 9:873829. 10.3389/fcvm.2022.873829 35811723 PMC9262125

[69] Frandsen S Kopp S Wehland M Pietsch J Infanger M Grimm D. Latest results for anti-angiogenic drugs in cancer treatment. Curr Pharm Des. (2016) 22:5927–42. 10.2174/1381612822666160715130419 27426129

[70] Shimoi T Sagara Y Hara F Toyama T Iwata H. First-line endocrine therapy for postmenopausal patients with hormone receptor-positive, HER2-negative metastatic breast cancer: a systematic review and meta-analysis. Breast Cancer. (2020) 27:340–6. 10.1007/s12282-020-01054-7 32043218 PMC7196086

[71] Tripathy D Im SA Colleoni M Franke F Bardia A Harbeck N. Ribociclib plus endocrine therapy for premenopausal women with hormone-receptor-positive, advanced breast cancer (MONALEESA-7): a randomised phase 3 trial. Lancet Oncol. (2018) 19:904–15. 10.1016/S1470-2045(18)30292-4 29804902

[72] Robert M Frenel JS Bourbouloux E Rigaud DB Patsouris A Augereau P. An Update on the Clinical Use of CDK4/6 Inhibitors in Breast Cancer. Drugs. (2018) 78:1353–62. 10.1007/s40265-018-0972-9 30143968

[73] Schoninger SF Blain SW. The Ongoing Search for Biomarkers of CDK4/6 Inhibitor Responsiveness in Breast Cancer. Mol Cancer Ther. (2020) 19:3–12. 10.1158/1535-7163.MCT-19-0253 31909732 PMC6951437

[74] Sammons S Shastry M Dent S Anders C Hamilton E. Practical treatment strategies and future directions after progression while receiving CDK4/6 inhibition and endocrine therapy in advanced HR(+)/HER2(-) breast cancer. Clin Breast Cancer. (2020) 20:1–11. 10.1016/j.clbc.2019.06.017 31780379

[75] Presti D Quaquarini E. The PI3K/AKT/mTOR and CDK4/6 pathways in endocrine resistant HR+/HER2- metastatic breast cancer: biological mechanisms and new treatments. *Cancers (Basel)* (2019) 11: Presti D, Quaquarini E. The PI3K/AKT/mTOR and CDK4/6 pathways in endocrine resistant HR+/HER2- metastatic breast cancer: biological mechanisms and new treatments. Cancers (Basel). (2019) 11 10.3390/cancers11091242 31450618 PMC6770492

[76] Cicini MP Ferretti G Morace N Nistico C Cognetti F Rulli F. Second-degree type 2 atrioventricular block requiring permanent cardiac pacing in patients on CDK4/6 inhibitors: report of two cases. Breast Care (Basel). (2022) 17:330–5. 10.1159/000519728 35957944 PMC9247538

[77] Shah A Bloomquist E Tang S Fu W Bi Y Liu Q. FDA Approval: ribociclib for the treatment of postmenopausal women with hormone receptor-positive, HER2-negative advanced or metastatic breast cancer. Clin Cancer Res. (2018) 24:2999–3004. 10.1158/1078-0432.CCR-17-2369 29437768

[78] Hortobagyi GN Stemmer SM Burris HA Yap YS Sonke GS Paluch-Shimon S. Updated results from MONALEESA-2, a phase III trial of first-line ribociclib plus letrozole versus placebo plus letrozole in hormone receptor-positive, HER2-negative advanced breast cancer. Ann Oncol. (2018) 29:1541–7. 10.1093/annonc/mdy155 29718092

[79] Tao YF Wang NN Xu LX Li ZH Li XL Xu YY. Molecular mechanism of G1 arrest and cellular senescence induced by LEE011, a novel CDK4/CDK6 inhibitor, in leukemia cells. Cancer Cell Int. (2017) 17:35. 10.1186/s12935-017-0405-y 28286417 PMC5340031

[80] Gintant GA Su Z Martin RL Cox BF. Utility of hERG assays as surrogate markers of delayed cardiac repolarization and QT safety. Toxicol Pathol. (2006) 34:81–90. 10.1080/01926230500431376 16507548

[81] Santoni M Occhipinti G Romagnoli E Miccini F Scoccia L Giulietti M. Different cardiotoxicity of palbociclib and ribociclib in breast cancer: gene expression and pharmacological data analyses, biological basis, and therapeutic implications. BioDrugs. (2019) 33:613–20. 10.1007/s40259-019-00382-1 31529317

[82] Eisenhauer EA Therasse P Bogaerts J Schwartz LH Sargent D Ford R. et al. New response evaluation criteria in solid tumours: revised RECIST guideline (version 11). Eur J Cancer. (2009) 45:228–47. 10.1016/j.ejca.2008.10.026 19097774

[83] Miles DW Chan A Dirix LY Cortes J Pivot X Tomczak P. Phase III study of bevacizumab plus docetaxel compared with placebo plus docetaxel for the first-line treatment of human epidermal growth factor receptor 2-negative metastatic breast cancer. J Clin Oncol. (2010) 28:3239–47. 10.1200/JCO.2008.21.6457 20498403

[84] Cella D Wang M Wagner L Miller K. Survival-adjusted health-related quality of life (HRQL) among patients with metastatic breast cancer receiving paclitaxel plus bevacizumab versus paclitaxel alone: results from Eastern Cooperative Oncology Group Study 2100 (E2100). Breast Cancer Res Treat. (2011) 130:855–61. 10.1007/s10549-011-1725-6 21874312 PMC3684040

[85] Franco TH Khan A Joshi V Thomas B. Takotsubo cardiomyopathy in two men receiving bevacizumab for metastatic cancer. Ther Clin Risk Manag. (2008) 4:1367–70. 10.2147/TCRM.S3960 19337443 PMC2643117

[86] Touyz RM Herrmann J. Cardiotoxicity with vascular endothelial growth factor inhibitor therapy. NPJ Precis Oncol. (2018) 2:13. 10.1038/s41698-018-0056-z 30202791 PMC5988734

[87] Khongorzul P Ling CJ Khan FU Ihsan AU Zhang J. Antibody-Drug Conjugates: A Comprehensive Review. Mol Cancer Res. (2020) 18:3–19. 10.1158/1541-7786.MCR-19-0582 31659006

[88] Makawita S Meric-Bernstam F. Antibody-Drug Conjugates: Patient and Treatment Selection. Am Soc Clin Oncol Educ Book. (2020) 40:1–10. 10.1200/EDBK_280775 32213087

[89] Leung D Wurst JM Liu T Martinez RM Datta-Mannan A Feng Y. Antibody conjugates-recent advances and future innovations. Antibodies (Basel). (2020) 9:2. 10.3390/antib9010002 31936270 PMC7148502

[90] Garcia-Alonso S Ocana A Pandiella A. Trastuzumab emtansine: mechanisms of action and resistance, clinical progress, and beyond. Trends Cancer. (2020) 6:130–46. 10.1016/j.trecan.2019.12.010 32061303

[91] Hunter FW Barker HR Lipert B Rothe F Gebhart G Piccart-Gebhart MJ. Mechanisms of resistance to trastuzumab emtansine (T-DM1) in HER2-positive breast cancer. Br J Cancer. (2020) 122:603–12. 10.1038/s41416-019-0635-y 31839676 PMC7054312

[92] Martinez MT Perez-Fidalgo JA Martin-Martorell P Cejalvo JM Pons V Bermejo B. Treatment of HER2 positive advanced breast cancer with T-DM1: A review of the literature. Crit Rev Oncol Hematol. (2016) 97:96–106. 10.1016/j.critrevonc.2015.08.011 26318092

[93] Barbieri MA Sorbara EE Cicala G Santoro V Cutroneo PM Franchina T. Adverse drug reactions with HER2-positive breast cancer treatment: an analysis from the Italian pharmacovigilance database. Drugs Real World Outcomes. (2022) 9:91–107. 10.1007/s40801-021-00278-z 34528216 PMC8844323

[94] Ponde N Ameye L Lambertini M Paesmans M Piccart M De Azambuja E. Trastuzumab emtansine (T-DM1)-associated cardiotoxicity: Pooled analysis in advanced HER2-positive breast cancer. Eur J Cancer. (2020) 126:65–73. 10.1016/j.ejca.2019.11.023 31923729

[95] Tolaney SM Tayob N Dang C Yardley DA Isakoff SJ Valero V. Adjuvant trastuzumab emtansine versus paclitaxel in combination with trastuzumab for stage I HER2-positive breast cancer (ATEMPT): a randomized clinical trial. J Clin Oncol. (2021) 39:2375–85. 10.1200/JCO.20.03398 34077270

[96] Barroso-Sousa R Tarantino P Tayob N Dang C Yardley DA Isakoff SJ. Cardiac outcomes of subjects on adjuvant trastuzumab emtansine vs paclitaxel in combination with trastuzumab for stage I HER2-positive breast cancer (ATEMPT) study (TBCRC033): a randomized controlled trial. NPJ Breast Cancer. (2022) 8:18. 10.1038/s41523-022-00385-2 35173164 PMC8850608

[97] Verma S Miles D Gianni L Krop IE Welslau M Baselga J. Trastuzumab emtansine for HER2-positive advanced breast cancer. N Engl J Med. (2012) 367:1783–91. 10.1056/NEJMoa1209124 23020162 PMC5125250

[98] Krop IE Kim SB Gonzalez-Martin A Lorusso PM Ferrero JM Smitt M. Trastuzumab emtansine versus treatment of physician's choice for pretreated HER2-positive advanced breast cancer (TH3RESA): a randomised, open-label, phase 3 trial. Lancet Oncol. (2014) 15:689–99. 10.1016/S1470-2045(14)70178-0 24793816

[99] Nielsen FC Van Overeem Hansen T Sorensen CS. Hereditary breast and ovarian cancer: new genes in confined pathways. Nat Rev Cancer. (2016) 16:599–612. 10.1038/nrc.2016.72 27515922

[100] Ohmoto A Yachida S. Current status of poly(ADP-ribose) polymerase inhibitors and future directions. Onco Targets Ther. (2017) 10:5195–208. 10.2147/OTT.S139336 29138572 PMC5667784

[101] Slade D. PARP. and PARG inhibitors in cancer treatment. Genes Dev. (2020) 34:360–94. 10.1101/gad.334516.119 32029455 PMC7050487

[102] Curtin NJ Szabo C. Poly(ADP-ribose) polymerase inhibition: past, present and future. Nat Rev Drug Discov. (2020) 19:711–36. 10.1038/s41573-020-0076-6 32884152

[103] Ledermann J Harter P Gourley C Friedlander M Vergote I Rustin G. Olaparib maintenance therapy in platinum-sensitive relapsed ovarian cancer. N Engl J Med. (2012) 366:1382–92. 10.1056/NEJMoa1105535 22452356

[104] Robson M Im SA Senkus E Xu B Domchek SM Masuda N. Olaparib for metastatic breast cancer in patients with a germline BRCA mutation. N Engl J Med. (2017) 377:523–33. 10.1056/NEJMoa1706450 28578601

[105] Yamaoka K Fujiwara M Uchida M Uesawa Y Muroi N Shimizu T. Comprehensive analysis of adverse events induced by PARP inhibitors using JADER and time to onset. Life (Basel). (2022) 12:1355. 10.3390/life12091355 36143391 PMC9504973

[106] Okazaki T Chikuma S Iwai Y Fagarasan S Honjo T A. rheostat for immune responses: the unique properties of PD-1 and their advantages for clinical application. Nat Immunol. (2013) 14:1212–8. 10.1038/ni.2762 24240160

[107] Kwa MJ Adams S. Checkpoint inhibitors in triple-negative breast cancer (TNBC): Where to go from here. Cancer. (2018) 124:2086–103. 10.1002/cncr.31272 29424936

[108] Smahel M. PD-1/PD-L1 blockade therapy for tumors with downregulated MHC class I expression. Int J Mol Sci. (2017) 18:1331. 10.3390/ijms18061331 28635644 PMC5486151

[109] Emens LA Cruz C Eder JP Braiteh F Chung C Tolaney SM. Long-term clinical outcomes and biomarker analyses of atezolizumab therapy for patients with metastatic triple-negative breast cancer: a phase 1 study. JAMA Oncol. (2019) 5:74–82. 10.1001/jamaoncol.2018.4224 30242306 PMC6439773

[110] Adams S Schmid P Rugo HS Winer EP Loirat D Awada A. Pembrolizumab monotherapy for previously treated metastatic triple-negative breast cancer: cohort A of the phase II KEYNOTE-086 study. Ann Oncol. (2019) 30:397–404. 10.1093/annonc/mdy517 30475950

[111] Loibl S Untch M Burchardi N Huober J Sinn BV Blohmer JU. A randomised phase II study investigating durvalumab in addition to an anthracycline taxane-based neoadjuvant therapy in early triple-negative breast cancer: clinical results and biomarker analysis of GeparNuevo study. Ann Oncol. (2019) 30:1279–88. 10.1093/annonc/mdz158 31095287

[112] Baroudjian B Arangalage D Cuzzubbo S Hervier B Lebbe C Lorillon G. Management of immune-related adverse events resulting from immune checkpoint blockade. Expert Rev Anticancer Ther. (2019) 19:209–22. 10.1080/14737140.2019.1562342 30572735

[113] Lyon AR Yousaf N Battisti NML Moslehi J Larkin J. Immune checkpoint inhibitors and cardiovascular toxicity. Lancet Oncol. (2018) 19:e447–e58. 10.1016/S1470-2045(18)30457-1 30191849

[114] Yang Y Wu Q Chen L Qian K Xu X. Severe immune-related hepatitis and myocarditis caused by PD-1 inhibitors in the treatment of triple-negative breast cancer: a case report. Ann Transl Med. (2022) 10:424. 10.21037/atm-22-1284 35530956 PMC9073793

[115] Palaskas N Lopez-Mattei J Durand JB Iliescu C Deswal A. Immune checkpoint inhibitor myocarditis: pathophysiological characteristics, diagnosis, and treatment. J Am Heart Assoc. (2020) 9:e013757. 10.1161/JAHA.119.013757 31960755 PMC7033840

[116] Armenian SH Lacchetti C Barac A Carver J Constine LS Denduluri N. Prevention and monitoring of cardiac dysfunction in survivors of adult cancers: American Society of clinical oncology clinical practice guideline. J Clin Oncol. (2017) 35:893–911. 10.1200/JCO.2016.70.5400 27918725

[117] Farolfi A Melegari E Aquilina M Scarpi E Ibrahim T Maltoni R. Trastuzumab-induced cardiotoxicity in early breast cancer patients: a retrospective study of possible risk and protective factors. Heart. (2013) 99:634–9. 10.1136/heartjnl-2012-303151 23349345

[118] Florido R Smith KL Cuomo KK Russell SD. Cardiotoxicity from human epidermal growth factor receptor-2 (HER2) targeted therapies. J Am Heart Assoc. (2017) 6:e006915. 10.1161/JAHA.117.006915 28939718 PMC5634312

[119] Perez EA Suman VJ Davidson NE Sledge GW Kaufman PA Hudis CA. Cardiac safety analysis of doxorubicin and cyclophosphamide followed by paclitaxel with or without trastuzumab in the North Central Cancer Treatment Group N9831 adjuvant breast cancer trial. J Clin Oncol. (2008) 26:1231–8. 10.1200/JCO.2007.13.5467 18250349 PMC4048960

[120] Romond EH Jeong JH Rastogi P Swain SM Geyer CE. Jr., Ewer MS, et al. Seven-year follow-up assessment of cardiac function in NSABP B-31, a randomized trial comparing doxorubicin and cyclophosphamide followed by paclitaxel (ACP) with ACP plus trastuzumab as adjuvant therapy for patients with node-positive, human epidermal growth factor receptor 2-positive breast cancer. J Clin Oncol. (2012) 30:3792–9. 10.1200/JCO.2011.40.0010 22987084 PMC3478574

[121] Dempsey N Rosenthal A Dabas N Kropotova Y Lippman M Bishopric NH. Trastuzumab-induced cardiotoxicity: a review of clinical risk factors, pharmacologic prevention, and cardiotoxicity of other HER2-directed therapies. Breast Cancer Res Treat. (2021) 188:21–36. 10.1007/s10549-021-06280-x 34115243

[122] Guenancia C Lefebvre A Cardinale D Yu AF Ladoire S Ghiringhelli F. Obesity as a risk factor for anthracyclines and trastuzumab cardiotoxicity in breast cancer: a systematic review and meta-analysis. J Clin Oncol. (2016) 34:3157–65. 10.1200/JCO.2016.67.4846 27458291 PMC5569689

[123] Collier P Phelan D Klein A. A test in context: myocardial strain measured by speckle-tracking echocardiography. J Am Coll Cardiol. (2017) 69:1043–56. 10.1016/j.jacc.2016.12.012 28231932

[124] Rhea IB Rehman S Jarori U Choudhry MW Feigenbaum H Sawada SG. Prognostic utility of blood pressure-adjusted global and basal systolic longitudinal strain. Echo Res Pract. (2016) 3:17–24. 10.1530/ERP-15-0037 27249810 PMC5323871

[125] Curigliano G Cardinale D Dent S Criscitiello C Aseyev O Lenihan D. Cardiotoxicity of anticancer treatments: epidemiology, detection, and management. CA Cancer J Clin. (2016) 66:309–25. 10.3322/caac.21341 26919165

[126] Cardinale D Sandri MT Colombo A Colombo N Boeri M Lamantia G. Prognostic value of troponin I in cardiac risk stratification of cancer patients undergoing high-dose chemotherapy. Circulation. (2004) 109:2749–54. 10.1161/01.CIR.0000130926.51766.CC 15148277

[127] Rangarajan V Chacko SJ Romano S Jue J Jariwala N Chung J. Left ventricular long axis function assessed during cine-cardiovascular magnetic resonance is an independent predictor of adverse cardiac events. J Cardiovasc Magn Reson. (2016) 18:35. 10.1186/s12968-016-0257-y 27266262 PMC4897936

[128] From AM Maleszewski JJ Rihal CS. Current status of endomyocardial biopsy. Mayo Clin Proc. (2011) 86:1095–102. 10.4065/mcp.2011.0296 22033254 PMC3203000

[129] Yu AF Yadav NU Lung BY Eaton AA Thaler HT Hudis CA. Trastuzumab interruption and treatment-induced cardiotoxicity in early HER2-positive breast cancer. Breast Cancer Res Treat. (2015) 149:489–95. 10.1007/s10549-014-3253-7 25552363 PMC4970316

[130] Suter TM Procter M Van Veldhuisen DJ Muscholl M Bergh J Carlomagno C. Trastuzumab-associated cardiac adverse effects in the herceptin adjuvant trial. J Clin Oncol. (2007) 25:3859–65. 10.1200/JCO.2006.09.1611 17646669

[131] Oliva S Cioffi G Frattini S Simoncini EL Faggiano P Boccardi L. Administration of angiotensin-converting enzyme inhibitors and beta-blockers during adjuvant trastuzumab chemotherapy for nonmetastatic breast cancer: marker of risk or cardioprotection in the real world? Oncologist. (2012) 17:917–24. 10.1634/theoncologist.2011-0445 22673631 PMC3399646

[132] Gulati G Heck SL Ree AH Hoffmann P Schulz-Menger J Fagerland MW. Prevention of cardiac dysfunction during adjuvant breast cancer therapy (PRADA): a 2 x 2 factorial, randomized, placebo-controlled, double-blind clinical trial of candesartan and metoprolol. Eur Heart J. (2016) 37:1671–80. 10.1093/eurheartj/ehw022 26903532 PMC4887703

[133] Boekhout AH Gietema JA Milojkovic Kerklaan B Van Werkhoven ED Altena R Honkoop A. Angiotensin II-receptor inhibition with candesartan to prevent trastuzumab-related cardiotoxic effects in patients with early breast cancer: a randomized clinical trial. JAMA Oncol. (2016) 2:1030–7. 10.1001/jamaoncol.2016.1726 27348762

[134] Pituskin E Mackey JR Koshman S Jassal D Pitz M Haykowsky MJ. Multidisciplinary Approach to Novel Therapies in Cardio-Oncology Research (MANTICORE 101-Breast): A Randomized Trial for the Prevention of Trastuzumab-Associated Cardiotoxicity. J Clin Oncol. (2017) 35:870–7. 10.1200/JCO.2016.68.7830 27893331

[135] Navidi M Phillips AW Griffin SM Duffield KE Greystoke A Sumpter K. Cardiopulmonary fitness before and after neoadjuvant chemotherapy in patients with oesophagogastric cancer. Br J Surg. (2018) 105:900–6. 10.1002/bjs.10802 29601082

[136] Foulkes SJ Howden EJ Bigaran A Janssens K Antill Y Loi S. Persistent impairment in cardiopulmonary fitness after breast cancer chemotherapy. Med Sci Sports Exerc. (2019) 51:1573–81. 10.1249/MSS.0000000000001970 30829962

[137] Okwuosa TM Ray RM Palomo A Foraker RE Johnson L Paskett ED. Pre-diagnosis exercise and cardiovascular events in primary breast cancer: women's health initiative. JACC CardioOncol. (2019) 1:41–50. 10.1016/j.jaccao.2019.08.014 34396161 PMC8352124

[138] Gilchrist SC Barac A Ades PA Alfano CM Franklin BA Jones LW. Cardio-oncology rehabilitation to manage cardiovascular outcomes in cancer patients and survivors: a scientific statement from the American Heart Association. Circulation. (2019) 139:e997–e1012. 10.1161/CIR.0000000000000679 30955352 PMC7603804

[139] Dolan LB Barry D Petrella T Davey L Minnes A Yantzi A. The cardiac rehabilitation model improves fitness, quality of life, and depression in breast cancer survivors. J Cardiopulm Rehabil Prev. (2018) 38:246–52. 10.1097/HCR.0000000000000256 28525464

[140] Dias A Claudino W Sinha R Perez CA Jain D. Human epidermal growth factor antagonists and cardiotoxicity-A short review of the problem and preventative measures. Crit Rev Oncol Hematol. (2016) 104:42–51. 10.1016/j.critrevonc.2016.04.015 27338847

